# Longitudinal Associations between Physical Pain and Older Adults’ Intergenerational Social Networks

**DOI:** 10.1093/geroni/igaf122.252

**Published:** 2025-12-31

**Authors:** Shelbie Turner, Carson De Fries, Carly Roman-Woo, Shannon Jarrott

**Affiliations:** Weill Cornell Medicine, New York, New York, United States; University of Utah, Salt Lake City, Utah, United States; Archstone Foundation, Long Beach, California, United States; The Ohio State University, Columbus, Ohio, United States

## Abstract

Older adults with chronic pain often struggle to maintain social connections and have reduced social networks. Intergenerational social relationships may be especially challenging to maintain in the presence of pain. Yet, limited scholarship characterizes the intergenerational social networks of older adults with pain, nor how pain impacts the prevalence of intergenerational social partners over time. To fill this gap, we analyzed cross-sectional and longitudinal associations between activity-limiting pain and intergenerational social networks among older adults participating in the National Health and Aging Trends Study (n = 2,348). Based on an existing evidence-based coding process, we coded the number of close social partners who were 10, 15, 20, and 25 years younger than the age of each participant. We then ran regression models to analyze whether activity-limiting pain was associated with the number of social partners in each intergenerational group (i.e., 10, 15, 20, and 25+ years younger). Models revealed that having activity-limiting pain was associated with fewer social partners 25 years younger than the participant cross-sectionally (-0.08, p = 0.02) and longitudinally two (-0.07, p = 0.02) and three (-0.08, p = 0.04) years later. There were no associations between activity-limiting pain and the number of social partners who were 10, 15, or 20 years younger than the participant. Pain appears to be particularly consequential to older adults’ relationships with people multiple generations younger than them (i.e., “skipped generation relationships”). Efforts to support older adults’ engagement with skipped generation social partners are necessary, and researchers should evaluate how improved intergenerational connectivity can support older adults’ pain management.

